# Threshold and Standard Error of Measurement for Relative Rest Time of Pericervical Muscles During Prolonged Computer-Typing Tasks in Individuals With Neck Symptoms

**DOI:** 10.7759/cureus.69119

**Published:** 2024-09-10

**Authors:** Kazuki Kikkawa, Hiroshi Takasaki

**Affiliations:** 1 Health and Social Services, Saitama Prefectural University, Koshigaya, JPN; 2 Physical Therapy, Saitama Prefectural University, Koshigaya, JPN

**Keywords:** computer task, electromyography, measurement error, minimum detectable change, neck symptoms, relative rest time

## Abstract

Introduction

The most common work-related musculoskeletal disorder worldwide is neck pain, especially among individuals who extensively use computers in a seated position. One biomedical cause of this neck pain is the prolonged activity of the muscles around the neck. Electromyography (EMG) has been used to evaluate the frequency and intensity of muscle activity. “Relative rest time (RRT)” is an index derived indicating the proportion of time below a certain threshold to the total task time. This study aimed to investigate the measurement errors and minimum detectable change (MDC) of RRT of the pericervical muscles during prolonged typing tasks in individuals with neck symptoms and to examine the differences in measurement errors at 3 µV and 6 µV thresholds.

Methods

This test-retest reliability study was conducted twice with a one-week interval to examine measurement errors of the RRT using surface EMG. The number of participants was set to 30 subjects who had neck symptoms with a Neck Disability Index of 16% or higher. The primary outcome measure was RRT of the following: the pericervical muscles of the right side during a 60-minute typing task; the splenius capitis muscle, upper trapezius (UT) muscle, middle trapezius muscle, sternocleidomastoid muscle, serratus anterior muscle, longissimus muscle, and pectoralis major muscle. RRT was calculated as the percentage of time that muscle activity was below the threshold for more than 0.250 seconds continuously during a 60-minute typing task. The standard error of measurement (SEM) and MDC were calculated with the two thresholds of 3 µV and 6 µV. The SEMs of the two thresholds were compared using a paired method.

Results

Ultimately, the data of 26 participants were analyzed. The SEM (MDC) values of the RRT at the 3 µV and 6 µV thresholds were 19.22 (53.27) and 9.52 (26.39) for the splenius capitis muscle, 3.24 (8.97) and 0.38 (1.05) for the sternocleidomastoid muscle, 15.47 (42.88) and 18.79 (52.08) for the UT muscle, 21.28 (58.99) and 2.28 (6.32) for the middle trapezius muscle, 13.67 (37.90) and 11.64 (32.27) for the serratus anterior muscle, 16.81 (46.60) and 3.32 (9.20) for the longissimus muscle, and 8.97 (24.87) and 4.24 (11.74) for the pectoralis major muscle, respectively. The SEMs of the RRT with the 6 µV threshold were statistically significantly lower than those with the 3 µV threshold in all pericervical muscles, except for the UT muscle.

Conclusion

This study identified the SEM and MDC of the RRT for the pericervical muscles during prolonged typing tasks in individuals with neck symptoms. Except for the UT muscle, the SEMs of the RRT with the 6 µV threshold were statistically smaller than those with the 3 µV threshold. Therefore, when using the RRT in intervention studies that aim to reduce muscle activity during typing in those with neck symptoms, the 6 µV threshold measurement would be recommended for the RRT of the pericervical muscles except for the UT muscle.

## Introduction

The most common work-related musculoskeletal disorder worldwide is neck pain, especially among individuals who extensively use computers in the seated position [1]. A possible biomedical cause of neck pain in computer users is a prolonged activity of the muscles around the neck, and electromyography (EMG) has been used to understand the magnitude of prolonged muscle activity.

Historically, gap frequency, which evaluates the frequency of muscle activity below a certain amplitude [2], has been developed and used in various studies. Notably, even small loads for extended periods of time can result in cumulative injuries. Therefore, gap frequency is suitable for assessing the load on muscles in prolonged low-load tasks, and it can predict neck/shoulder disorder development [3]. Relative rest time (RRT), which is the ratio of the time below a certain threshold to the total task time, was derived from gap frequency. Hermens and Vollenbroek-Hutten [4] recommended RRT for muscle activity dynamic assessment between sessions because it is less sensitive to differences in electrode position.

The threshold for RRT can be either normalized across participants on the basis of maximum voluntary contraction or relative voluntary contraction (RVC) or standardized across participants using a uniform value. Hansson et al. [5] recommended a threshold for RRT in the upper trapezius (UT) muscle of 0.3-1% of the maximum voluntary contraction, which corresponded to 2.6-8.9 µV. Subsequently, using a normalized threshold for the UT muscle did not reduce intrasubject variability any more than using a uniform value among healthy individuals [4], and measurement error was less with a uniform threshold of 3 µV or 6 µV than with normalized thresholds using RVC [6]. Furthermore, studies have suggested that RVC measurements in those with neck symptoms can be unreliable [7]. Therefore, to date, RRT using a uniform threshold for all participants appears to be the most promising method for muscle activity dynamic assessment during prolonged computer tasks in individuals with neck symptoms. Delisle et al. [6] investigated the standard error of measurement (SEM) of RRT for the UT muscle during a prolonged computer task in mixed samples of individuals with and without neck pain and recommended using a uniform 3 µV or 6 µV threshold, where the minimum threshold was 3 µV, considering the difficulty in determining whether signals below a 3 µV threshold indicate muscle activity or background noise.

Prolonged computer work could result in postural changes from the neck to the upper trunk, which is characteristic of a forward head posture and may be accompanied by changes in the muscle activity dynamics of the pericervical muscles. To date, the SEM of RRT for the pericervical muscles other than the UT muscle has not been investigated in individuals with neck symptoms. Furthermore, whether the SEM is different in the UT muscle at the 3 µV or 6 µV threshold has never been statistically evaluated. This study aimed to investigate the measurement errors of the RRT of the pericervical muscles during a prolonged typing task in individuals with neck symptoms and examine whether the measurement error was different at the 3 µV and 6 µV thresholds.

## Materials and methods

Design

To examine RRT measurement errors using surface EMG, this test-retest reliability study was conducted over one week. The Research Ethics Committee of Saitama Prefectural University approved this study (approval number: 22502).

Participants

Participants were recruited via campus email between April 2022 and December 2022. To account for the possibility of missing data, the number of participants was set to 30, according to the sample size of a previous study (n=27) [6]. The following were the inclusion criteria: (1) aged 18-60 years, (2) neck symptoms including stiff neck/shoulders with a Neck Disability Index (NDI) of 16% or higher [8], and (3) at least two days/week of computer use for two hours or more per week [9]. The following were the exclusion criteria: (1) a history of whiplash, neck fracture, or surgery; (2) inability to continuously sit at the edge of the laboratory environment for one hour for any reason; (3) difficulty with typing activities for any reason; and (4) inability to perform the standard EMG preparation for any reason (e.g., skin inflammation or trauma).

To understand the characteristics of the participants in addition to age, sex, dominant hand, body mass index, symptom location [10], and symptom duration, the following patient-reported outcome measures (PROMs) were assessed: NDI, four-item pain intensity measure (P4), and EuroQol-5 Dimensions (EQ-5D).

The NDI is a PROM developed for assessing disability in individuals with neck pain and has been widely used for assessing neck pain-associated disability [11]. Considering that the Japanese version set a cutoff of 15% for the degree of disability sufficient to require a visit to a medical facility [8], we adopted a value of 16% or higher as our inclusion criterion to increase the generalizability of the findings of our study, which included participants from a university.

P4 is a PROM and a reliable pain intensity measure, with higher total scores indicating greater pain intensity (0-40). The minimum detectable change (MDC) is 9.1 points [12], which we used as the criterion for comparing the participant’s status between the two measurement sessions.

The EQ-5D is a PROM that assesses quality of life and comprises five items and five statements regarding the quality of life. Higher scores (0-1) indicate a better quality of life [13].

Procedures

RRT of the following pericervical muscles of the right side was measured during a 60-minute typing task: splenius capitis, UT, middle trapezius, sternocleidomastoid, serratus anterior, longissimus, and pectoralis major muscles. Participants sat on a chair that was adjusted to have a backrest angle of 110°. Considering our plan to conduct a subsequent study to examine the effects of a lumbar spine roll on pericervical muscles, a backrest angle of 110° was selected on the basis of a study suggesting that such an angle promoted the greatest change in neck alignment using a lumbar roll [14]. The posture at the start of typing was adjusted so that the hip, knee, and elbow joints were each at 90°, and the height of the monitor was adjusted to eye level [9].

As a baseline, for the first minute of data collection, participants maintained a relaxed sitting posture (resting position) with their hands on an external keyboard (TK-FCM077PBK; Elecom, Osaka, Japan). According to the procedure of a previous study [9], using Bruce’s Typing Wizard software (Roseland Productions, New York, NY, USA), participants typed for 60 minutes at a comfortable pace, displaying the document to be typed. Errors were ignored, and no instructions were provided regarding typing posture.

Following the Surface EMG for the Non-invasive Assessment of Muscles (SENIAM) recommendations for skin preparation, self-adhesive Ag/AgCl electrodes (ECG electrodes 2009111-150; CareFusion, Helsinki, Finland) were attached to the assessed muscles as per Criswell [15] with a 20 mm interelectrode distance (Table 1). Muscle activity was measured using a surface EMG device (myoMUSCLE™ system; Noraxon USA, Inc., Scottsdale, AZ, USA) with a 1,500 Hz sampling frequency (digital resolution, 16 bit; band-pass filter, 10-500 Hz; input impedance, more than 100 Ω; common mode rejection ratio, more than 100 dB; electric gain, 200; and overall gain, 500). The amplitude of muscle activity was evaluated by reducing the electrocardiogram complex, filtering the EMG data with a 20-500 Hz bandpass filter [16], and calculating the root mean square (RMS) with a 100 millisecond sliding window. RRT was calculated as the percentage of time that muscle activity was below the threshold for more than 0.250 seconds continuously during a 60-minute typing task, according to a previous study [6]. The two thresholds of 3 µV and 6 µV, which were recommended by Delisle et al. [6], were examined.

**Table 1:** Electrode locations

Muscle (right side)	
Splenius capitis muscle	2 cm right lateral from the spinous process of the fourth cervical vertebrae electrode on the cephalic side
Upper trapezius muscle	medial electrode midway between the spinous process of the seventh cervical vertebra and the right acromion
Middle trapezius muscle	2 cm right lateral from the spinous process of the third thoracic vertebra electrode on the cephalic side
Sternocleidomastoid muscle	halfway between the mastoid process and the sternal notch, running parallel to the muscle fibers
Serratus anterior muscle	horizontally just below the axillary area, at the level of the inferior tip of the scapula, and medial to the latissimus dorsi
Longissimus muscle	2 cm right lateral from the third lumbar spinous process is the cephalad electrode
Pectoralis major muscle	2 cm inside from the right axilla is the outer electrode

Statistical analysis

SEM was calculated using the following formula [17]:

$SEM = SD_{pool}\sqrt{1 - ICC}$

where intraclass correlation coefficients (ICCs) were calculated on the basis of a two-way mixed-effects mode (ICCs(3,1)) according to the ICC guidelines [18]. SDpool was calculated using the standard deviations SDs of datasets at the two measurement sessions (e.g., the SD of the dataset at the first measurement session, SD_1_) using the following formula:

$SD_{pool}=\sqrt{\frac{{SD_1^{2}}+{SD_2^{2}}}{2}}$

Furthermore, MDCs were calculated using the following formula:

$MDC = SEM \times 1.96 \times \sqrt{2 \phantom{0}}$

The ICC is a measure of relative reliability and indicates the degree of repeatability by the variability of the data [18]. As opposed to the ICC, the MDC is a measure of absolute reliability and represents a magnitude of change that reflects a true change in performance, beyond what can be accounted for by measurement error or random variability [19]. The absolute reliability provides us with more information than the relative reliability, and MDCs in this study would allow us to consider the extent to which RRT can be utilized in future studies that examine changes in muscle activity during computer tasks through ergonomic interventions. Hence, the MDC was used for the interpretation of reliability according to a previous study [20]: MDC of <10%, excellent reliability, and MDC of 10-30%, acceptable reliability.

To statistically verify whether a difference in the SEM exists between the 3 µV and 6 µV thresholds, a paired method was used [21]. First, the difference (D) in RRT between the first and second sessions was calculated for each of the 3 µV and 6 µV thresholds. The dataset of the sum of the Ds with the two thresholds (e.g., D in Participant 1 with a 3 µV threshold [D_1-3µV_] + D in Participant 1 with a 6 µV threshold [D_1-6µV_], D_2-3µV_ + D_2-6µV_, D_3-3µV_ + D_3-6µV_, …, D_30-3µV_ + D_30-6µV_) and the dataset of the difference of the D (e.g., D_1-3µV_ - D_1-6µV_, D_2-3µV_ − D_2-6µV_, D_3-3µV_ − D_3-6µV_, …, D_30-3µV_ − D_30-6µV_) for the same participant were calculated. Pearson’s correlation coefficients were calculated between the two datasets. When a statistically significant correlation was identified, a statistically significant difference in the SEMs from the two RRT thresholds was detected.

A paired t-test was performed on the mean RMS values at rest for one minute before task initiation to confirm the comparability of the measurement environment between the two sessions. SPSS Statistics version 25.0 (IBM Corp. Released 2017. IBM SPSS Statistics for Windows, Version 25.0. Armonk, NY: IBM Corp.) was performed for all statistical analyses. Statistical significance was set at a value of 0.05.

## Results

**Figure 1:**
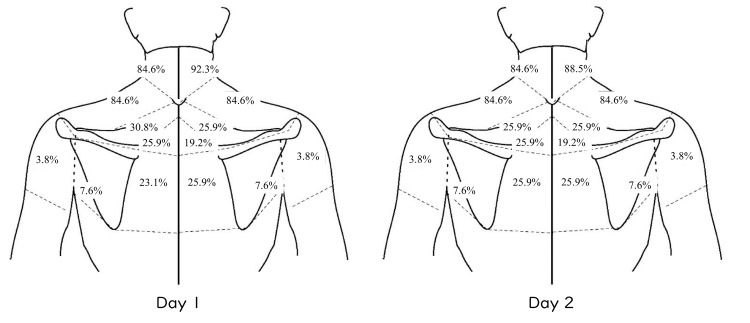
Symptom distributions Percentage (%) of symptom distribution in 26 participants. Image Credit: Authors

Thirty individuals participated in the first measurement. However, three individuals were unable to participate in the second measurement because of illness. The same measurement environment was not prepared for institutional issues for one participant, and the data of this participant were excluded from the analysis. Among the 26 participants who completed the two measurements, none had a change in P4 of more than 10 between sessions. Therefore, the data of 26 participants were analyzed. Table 2 presents the demographic data of 26 participants. Symptom locations are summarized in Figure 1.

**Table 2:** Participants demographics (N=26) Values are presented with mean ± SD or number (%). BMI: body mass index, P4: 4-item pain intensity measure, NDI: Neck Disability Index, EQ-5D: EuroQol-5 dimensions

Demographics	Value	
Male	7 (26.9)	
Female	19 (73.1)	
Age (years)	23.3 ± 7.5	
BMI (kg/m^2^)	21.1 ± 3.3	
Dominant arm left	0 (0)	
Dominant arm right	26 (100)	
Symptom duration	12.9 ± 24.5	
	Day 1	Day 2
P4 (0-40)	15.34 ± 6.4	15.4 ± 6.2
NDI (%)	26.2 ± 11.8	25.3 ± 9.8
EQ-5D (0-1)	0.7 ± 0.1	0.7 ± 0.1

No statistically significant difference (all p>0.05) was noted in the mean RMS of all muscles at rest for one minute before task initiation (Table 3). In all muscles, the upper limit of the 95% confidence interval (CI) of the mean value was less than 2 µV.

**Table 3:** Mean RMS and p-value of all muscles at rest for one minute before task initiation Values are presented with a mean RMS (95% CI) at rest for one minute before task initiation (µV). Statistical significance was set at a value of 0.05. RMS: root mean square, CI: confidence interval

Muscle	Day 1	Day 2	p-value
Splenius capitis muscle	0.43 (0.35-0.51)	0.52 (0.38-0.67)	0.224
Sternocleidomastoid muscle	0.37 (0.29-0.45)	0.40 (0.29-0.51)	0.642
Upper trapezius muscle	1.14 (0.66-1.62)	1.19 (0.72-1.66)	0.765
Middle trapezius muscle	0.83 (0.57-1.09)	0.77 (0.62-0.92)	0.572
Serratus anterior muscle	0.48 (0.31-0.65)	0.47 (0.32-0.62)	0.925
Longissimus muscle	0.75 (0.57-0.94)	0.80 (0.52-1.08)	0.692
Pectoralis major muscle	0.44 (0.21-0.68)	0.46 (0.21-0.71)	0.858

The mean RRT at each threshold and its 95% CIs are shown in Table 4. The mean RRTs in all of the pericervical muscles, except for the UT muscle, exceeded 80% at both thresholds.

**Table 4:** Means of the RRT Values are presented with a mean (95% CI) of RRT during a 60-minute typing task (%). RRT: relative rest time, CI: confidence interval

	Day 1		Day 2	
Muscle	3 µV threshold	6 µV threshold	3 µV threshold	6 µV threshold
Splenius capitis muscle	99.37 (98.99-99.79)	99.95 (99.88-100.02)	98.69 (97.58-99.81)	99.91 (99.83-99.99)
Sternocleidomastoid muscle	99.67 (99.50-99.84)	99.93 (99.88-99.98)	99.63 (99.44-99.82)	99.93 (99.89-99.97)
Upper trapezius muscle	56.40 (41.19-71.61)	70.30 (56.07-84.52)	59.70 (43.82-75.58)	76.50 (63.07-89.93)
Middle trapezius muscle	90.33 (81.46-99.20)	99.09 (98.38-99.80)	91.11 (83.59-98.64)	99.36 (98.87-99.86)
Serratus anterior muscle	98.55 (97.57-99.54)	99.62 (99.19-100.16)	98.59 (97.32-99.86)	99.73 (99.27-100.20)
Longissimus muscle	83.91 (72.35-95.47)	97.87 (95.64-100.10)	81.37 (68.95-93.80)	98.55 (96.46-100.63)
Pectoralis major muscle	95.35 (92.18-98.53)	99.28 (98.62-99.94)	95.03 (91.74-98.32)	98.13 (96.36-99.90)

The SEM values for each muscle at the two thresholds and the statistical results in the paired method on the SEM are presented in Table 5. The SEM values of RRT for the UT muscle were not statistically different. In other muscles, the SEMs of RRT with the 6 µV threshold were statistically smaller (all p<0.05) than those with the 3 µV threshold.

**Table 5:** SEM of RRT and p-value of a difference in SEM between 3 µV and 6 µV Values are presented with an SEM of RRT during a 60-minute typing task (%). Statistical significance was set at a value of 0.05. SEM: standard error of measurement, RRT: relative rest time

Muscle	3 µV threshold	6 µV threshold	p-value
Splenius capitis muscle	19.22	9.52	<0.001
Sternocleidomastoid muscle	3.24	0.38	<0.001
Upper trapezius muscle	15.47	18.79	0.13
Middle trapezius muscle	21.28	2.28	<0.001
Serratus anterior muscle	13.67	11.64	<0.001
Longissimus muscle	16.81	3.32	<0.001
Pectoralis major muscle	8.79	4.24	<0.001

The MDC for each muscle is shown in Table 6. The reliability of RRTs with a 3 µV threshold in the sternocleidomastoid and pectoralis major muscles and a 6 µV threshold in the splenius capitis, sternocleidomastoid, middle trapezius, longissimus, and pectoralis major muscles can be interpreted as acceptable.

**Table 6:** Ninety-five percent CI of the MDC in RRT Values are presented with a 95% CI of the MDC in RRT during a 60-minute typing task (%). * excellent reliability, <10%, † acceptable reliability, 10-30%, CI: confidence interval, MDC: minimum detectable change, RRT: relative rest time

Muscle	3 µV threshold	6 µV threshold
Splenius capitis muscle	53.27	26.39^†^
Sternocleidomastoid muscle	8.97*	1.05*
Upper trapezius muscle	42.88	52.08
Middle trapezius muscle	58.99	6.32*
Serratus anterior muscle	37.90	32.27
Longissimus muscle	46.60	9.20*
Pectoralis major muscle	24.87^†^	11.74^†^

## Discussion

To the best of our knowledge, this is the first study to examine the RRT thresholds of the pericervical muscles during a prolonged typing task among individuals with neck symptoms and determine RRT measurement errors. Investigating the effects of postural improvement in patients with nonspecific neck pain has been a research priority in recent years [22], and EMG has the potential as an objective outcome of an intervention. Intervention studies have recently recommended the use of 95% CIs of mean differences and minimal clinically important differences (MCIDs) rather than statistical significance for estimating intervention effects [23]. Therefore, the identification of MDC in RRT, which corresponds with the MCIDs in the experimental study, will allow for high-quality clinical trials on the physical effects of postural interventions in patients with nonspecific neck pain.

Considerations for the use of the RRT in future studies

Regarding reliability, acceptable reliability was not detected in the UT muscle and serratus anterior muscle regardless of the two measurement thresholds. SEMs were statistically smaller with the 6 µV threshold measurement than the 3 µV threshold, and at least acceptable reliability was detected in all pericervical muscles, except for the UT muscle and serratus anterior muscle. Therefore, the 6 µV threshold measurement would be more highly recommended than the 3 µV threshold measurement for the RRT of the pericervical muscles except for the UT muscle and serratus anterior muscle. On the other hand, these results do not mean that studies using RRTs of the UT muscle and serratus anterior muscle should not be conducted. It may still be possible to use either threshold for the UT muscle and the 6 µV threshold for the serratus anterior muscle to measure the impact of ergonomic interventions on muscle activity during computer tasks. However, when such a verification is conducted, it is necessary to consider whether the intervention is expected to be effective beyond each MDC.

A previous study reported that the SEMs of RRT values of the UT muscle using the 3 µV threshold were 7% and 14% for 15-minute and 45-minute computer tasks, respectively [6], and the value was 15.48% in the current study, indicating similar results [6]. Conversely, as individuals with neck pain may have higher resting muscle activity than healthy individuals [24], the RRT of the UT muscle in the current study was expected to be lower than the 20% RRT at a 3 µV threshold shown in a previous study [6]. However, in this study, higher RRT values were obtained with both thresholds than those in a previous study [6], ranging from 40% to 80% at a 3 µV threshold and from 50% to 90% at a 6 µV threshold. Therefore, RRT using a threshold of 6 µV may be able to identify a greater amount of change than 3 µV for validating the effectiveness of ergonomic interventions aimed at increasing muscle rest.

Comparisons to previous findings

Regarding the RRT values of the UT muscle, a previous study reported an RRT of 20% [6]; however, it was approximately 60% in the present study. There are several possible reasons for this difference. The first may be related to the study participants’ degree of disability. In the current study, individuals with neck symptoms were recruited to a university. A possible criticism is that a sample obtained using such a sampling method does not reflect the actual clinical population. Therefore, we used an NDI of 16% as the inclusion criterion [8], which has been reported as the cutoff value at which Japanese people with neck pain seek medical care. Furthermore, the participants in this study did not only have neck pain but also had a mixture of stiff neck and shoulders. Those with stiff shoulders may perceive their pain as less severe than those with neck pain [25]. As muscle activity increases with the degree of pain [26], the presence of a mixed sample of neck pain and stiff neck/shoulders may have influenced the higher RRT in this study than that in the previous study [6]. Second, the effects of the different task and measurement environments in the previous and present studies may have been stronger than the subject factor. For example, the previous study [6] required 15 or 45 minutes of typing and mouse control, whereas the present study required 60 minutes of typing. Samani et al. [27] also reported an average of 10 µV RMS during computer tasks using a mouse, and the side where the mouse was being operated had a higher UT muscle activity than the side where the mouse was not being operated [28]. Furthermore, in the present study, the chair, display, and starting limb positions were specified and standardized across participants, whereas the previous study [6] did not describe the details of the measurement environment, which may have influenced the results. Lastly, there may have been effects of differences in the electrodes and other measurement devices used (two silver bars (spaced 10 mm, each 10 mm in length and 1 mm in width) and the 512 Hz sampling rate) in the previous study [6].

Limitations

This study had some limitations. First, standard skin preparation was performed, but skin impedance was not checked in this study. Therefore, the potential resistance of the skin and subcutaneous tissue may have affected the RRT values and measurement error. In future studies, verifying skin impedance may allow for more accurate measurements. Second, there is a limitation in generalizability. In this study, participants were recruited based on the degree of disability caused by neck symptoms rather than the intensity of pain and from a university, resulting in the participation of young adults. Therefore, the results may differ in different populations in terms of symptoms or in children or older populations. Third, the electrode placement position might have affected the results. In this study, the electrodes were attached according to Criswell [15], and the RRT was greater than 80% in muscles other than the UT muscle. However, Falla et al. [29] recommended a slightly different electrode location to measure the EMG of the sternocleidomastoid muscle. When electrodes are attached to more sensitive locations, lower RRT values might be obtained. However, Falla et al. [29] suggested a sensitive electrode location using isometric contraction with a limited sample size of 11. Therefore, the certainty of the evidence may be unclear. Further, it has been reported that electrode position was not sensitive to differences in RRT for assessing the load on muscles during low-load tasks [4]. Therefore, it is unclear if the impact of the difference in the electrode locations between the current study and the previous study [29] on the RRT during the prolonged typing task is clinically meaningful. Furthermore, the electrode location on the longissimus muscle differed from the position recommended by SENIAM. However, there is a study indicating that the level of the lumbar spine at which the electrode is attached does not affect the EMG results [30]. Therefore, the difference in the electrode locations for the longissimus muscle may be negligible in the RRT. Further studies are required to investigate the impact of the difference in electrode locations on the RRT of a prolonged low-load task. Fourth, the SEM is affected by sample size. Although the sample size in the current study (n=26) was similar to that in a previous study (n=27) [6], the SEM and MDC would be smaller when the sample size is larger than the current study.

## Conclusions

This study investigated the SEM and MDC of RRT of the pericervical muscles during prolonged typing tasks in individuals with neck symptoms and examined the differences in SEM between each threshold. The SEM values of RRT for the UT muscle were similar to those in the previous study and were not statistically different between the 3 µV and 6 µV thresholds in this study. In other muscles, the SEMs of RRT with the 6 µV threshold were statistically smaller than those with the 3 µV threshold. Further, for splenius capitis, sternocleidomastoid, middle trapezius, longissimus, and pectoralis major muscles, the reliability was rated as acceptable by the MDC for both or either of the two threshold evaluations. Therefore, when using RRT in intervention studies aimed at reducing muscle activity during typing in those with neck symptoms, the 6 µV threshold measurement would be recommended for the RRT of the splenius capitis, sternocleidomastoid, middle trapezius, longissimus, and pectoralis major muscles. Care is needed for the RRT of the UT muscle and the serratus anterior muscle.
